# Elevated MED28 expression predicts poor outcome in women with breast cancer

**DOI:** 10.1186/1471-2407-10-335

**Published:** 2010-06-28

**Authors:** Nam K Yoon, Erin L Maresh, Yahya Elshimali, Ai Li, Steve Horvath, David B Seligson, David Chia, Lee Goodglick

**Affiliations:** 1 Department of Pathology and Laboratory Medicine, David Geffen School of Medicine at UCLA, Los Angeles, California, 90095, USA; 2 Department of Biostatistics, David Geffen School of Medicine at UCLA, Los Angeles, California, 90095, USA; 3 Department of Human Genetics, David Geffen School of Medicine at UCLA, Los Angeles, California, 90095, USA; 4 Jonsson Comprehensive Cancer Center, David Geffen School of Medicine at UCLA, Los Angeles, California, 90095, USA

## Abstract

**Background:**

MED28 (also known as EG-1 and magicin) has been implicated in transcriptional control, signal regulation, and cell proliferation. MED28 has also been associated with tumor progression in *in vitro *and *in vivo *models. Here we examined the association of MED28 expression with human breast cancer progression.

**Methods:**

Expression of MED28 protein was determined on a population basis using a high-density tissue microarray consisting of 210 breast cancer patients. The association and validation of MED28 expression with histopathological subtypes, clinicopathological variables, and disease outcome was assessed.

**Results:**

MED28 protein expression levels were increased in ductal carcinoma *in situ *and invasive ductal carcinoma of the breast compared to non-malignant glandular and ductal epithelium. Moreover, MED28 was a predictor of disease outcome in both univariate and multivariate analyses with higher expression predicting a greater risk of disease-related death.

**Conclusions:**

We have demonstrated that MED28 expression is increased in breast cancer. In addition, although the patient size was limited (88 individuals with survival information) MED28 is a novel and strong independent prognostic indicator of survival for breast cancer.

## Background

In 2008, an estimated 40,000 women died of breast cancer and over 190,000 women were newly diagnosed with the disease, making breast cancer the second leading cause of cancer death in women [1]. Although treatment based on the molecular characteristics of breast cancer subtypes has helped improve prognosis, much progress needs to be made (reviewed in [2-4]). The characterization of novel markers to augment our understanding of cancer and our ability to predict patient outcomes will greatly improve breast cancer management. A recently identified marker, MED28 (also known as EG-1 or magicin), has been found to be increased in breast cancer and may play a role in the progression of the disease [5-9].

MED28 is a 178 amino acid, ~24 kDa protein that was first identified as being differentially expressed in endothelial cells exposed to conditioned media from tumor cells [5,6]. Although the exact function of MED28 is unknown, it has been identified as one of approximately 30 subunits within the mammalian Mediator complex, which regulates activation and repression of RNA polymerase II transcribed genes [10-13]. In addition, MED28 has been found to be a binding partner for merlin, a cytoskeleton-related tumor suppressor important in neurofibromatosis 2 development [12,14].

Clues as to functional consequences of MED28 expression have been found in tumor model systems. The presence of MED28 has been shown to increase cellular proliferation in both cell culture and mouse xenograft models using human breast cancer cells [7]. Inhibition of MED28 expression by either siRNA or anti-MED28 antibody decreases cellular proliferation *in vitro *and *in vivo *[9]. Finally, in retrospective studies on human tissue samples, MED28 has been found to be up-regulated in breast, prostate, and colon cancers [6].

Here we examine the expression levels of MED28 on a population basis using a human breast cancer tissue microarray (TMA). Our findings show that MED28 expression is a significant independent predictor of survival in women with both early and late stage breast cancer.

## Methods

### Characteristics of the Breast TMA

#### Surgical cases represented on the TMA

A high-density breast TMA was constructed and utilized as previously described [15-17] with appropriate oversight by the UCLA Institutional Review Board. Briefly, the TMA was built using cores from archived formalin-fixed, paraffin-embedded breast tissue samples from 242 cases of patients who underwent surgery at the UCLA Medical Center between 1995 and 2000. Of these 242 cases, 213 women had surgery for suspected breast cancer while 29 women had breast reduction surgery. A "case" is defined as a surgery for which tissue was removed and could be used to construct the TMA. Of the women who had surgery for suspected breast cancer, 134 individuals had their primary surgery at UCLA. An additional 79 women came to the UCLA Medical center for a secondary follow-up surgery.

The spectrum of overall case histologies from the 213 patients who had surgery due to suspected cancer were as follows: 179 cases with invasive breast cancer histology (this was sub-divided into 122 cases which contained both invasive and *in situ *tumor histologies, and 57 cases which had invasive tumor histology alone); *in situ *tumor histology alone (22 cases); and individuals who had suspected cancer but who, upon surgery, were found to have ductal hyperplasia, atypical ductal hyperplasia, atypical lobular hyperplasia, or intraductal papilloma (4, 1, 2, and 5 cases respectively). Within the patients with invasive breast cancer (179 cases), 72 cases were associated with metastases. Forty-nine of these patients presented with metastasis at their first surgery (48 lymph node metastases, 1 distant metastasis).

#### Characteristics of spots on the TMA

At least three samples (cores) of each histology were taken to represent a given histology in each case. In total, the TMA consisted of 2,039 cores of which 924 were readable. Note that unreadable spots primarily included those that contained only stroma or fat or those that had fallen off during processing. The breakdown by core histology was 506 invasive tumors (440 invasive ductal carcinoma (IDC), 66 other breast cancer variants including invasive lobular carcinoma, invasive tubular carcinoma, apocrine carcinoma, mixed invasive ductal and lobular carcinoma, and medullary carcinoma), 98 *in situ *tumors (92 DCIS + 6 LCIS), 110 metastatic lesions, 14 atypical hyperplasia, 39 ductal hyperplasia, 109 normal matched tissues, 21 benign tissues, and 27 cores from breast reduction cases.

#### Case inclusion and exclusion criteria for outcomes analyses

**Figure 1:**
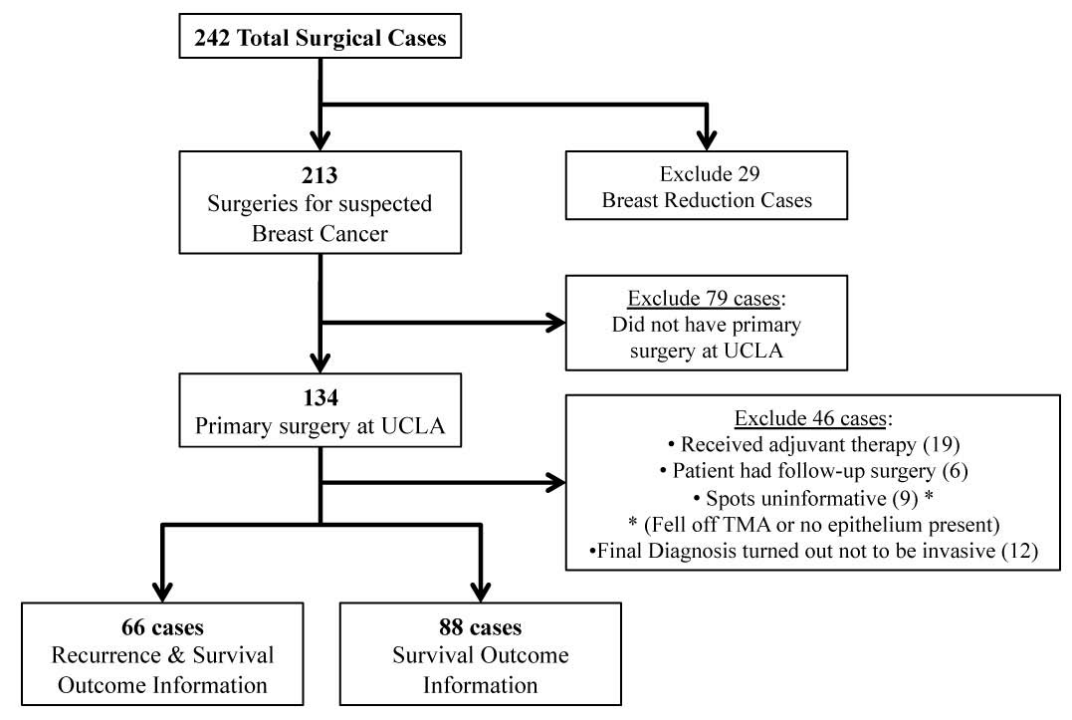
**Inclusion and exclusion criteria for outcome analyses**. The flow chart shows which cases were included in recurrence and survival analyses.

Only primary surgical cases of patients who did not receive neoadjuvant therapy were used for outcome analyses. The criteria for inclusion and exclusion of cases are shown in Figure 1. Briefly, of the 134 individuals who had primary surgery for suspected breast cancer at the UCLA Medical Center, 46 cases were excluded as shown in Figure 1, leaving 88 and 66 women for which we had i) survival or ii) recurrence and survival outcome information, respectively.

### Immunohistochemistry

The breast TMA was evaluated for MED28 expression using a standard immunohistochemistry protocol as previously described [6,15-17]. Briefly, 4 μm thick TMA sections were cut, deparaffinized, treated for antigen retrieval with 10 mM sodium citrate, pH 6 (95°C for 20 min), quenched for endogenous peroxidase activity, and blocked with 5% horse serum before incubation for 30 minutes with anti-MED28 primary antibody at a 1:300 dilution. The primary antibody was a polyclonal rabbit anti-human-MED28 antibody produced in the laboratory of Dr. Mai Brooks [6]. The primary antibody was detected by applying a horse anti-rabbit HRP secondary antibody and an avidin-biotin complex (Vector Laboratories, Burlingame, CA) followed by diaminobenzidine. Negative controls included primary incubation with preimmune rabbit serum. Her-2/neu status was determined by immunohistochemistry using the Hercep Test guidelines (DAKO, catalog K5204, Carpinteria, CA).

### TMA Scoring

Semiquantitative evaluation of MED28 staining was performed by a pathologist who tabulated the percentage of glandular cells that exhibited cytoplasmic staining at each intensity, from 0 to 3 (0 being below the level of detection, 1 being weak, 2 being moderate and 3 representing highest expression) as previously described [15-18]. Briefly, an integrated value was used to account for frequency and intensity of staining for each spot. The following formula was used to calculate this integrated value: [3(%x) + 2(%y) + 1(%z)]/100, where x, y, and z represent the percentage of cells staining at intensity 3, 2, and 1, respectively. Survival analyses were analyzed with patient case data. Case data was analyzed by using pooled expression results as previously described [16-19].

### Statistical Analyses

Individual integrated intensity measures for each spot were used for spot-level analysis, while the mean integrated intensity values for each case were used for case-level analysis as previously described [16-20]. Non-parametric two-group and multi-group comparisons were performed using the Mann-Whitney and Kruskal-Wallis tests, respectively. Non-parametric correlative analyses were performed using the Spearman correlation. Evaluation of MED28 and other clinico-pathological variables was performed using a Cox proportional hazards model. Patients were dichotomized at the 75^th ^percentile of MED28 expression for the whole cohort (defined high and low expression is shown in Table 1). Survival was visualized via Kaplan-Meier curves, and survival differences were tested using the log-rank test [16-20]. The statistical independence and significance of MED28 along with covariates were evaluated in a multivariate Cox model. All statistical analyses were performed using StatView Version 5.0 (SAS Institute, Cary, NC) or with the freely available software package, R http://www.r-project.org.

**Table 1:** Clinico-pathologic characteristics and MED28 expression in individuals with breast cancer

		MED28 Expression		Dichotomized		Continuous
		Low	High	P-Value	± σ	P-Value
All Invasive Patients	N = 88	N = 66	N = 22		0.946 ± 0.668	
Age at Diagnosis		= 55.2	= 55.9	0.8926^a^	ρ = 0.024^b^	0.8195^b^
Median (Range)	53 (30 - 89)	53.5 (30 - 89)	52 (36 - 89)			
25th to 75th Quartile	45 - 66	45 - 74	45 - 77.2			
Clinical Stage		= 1.712	= 2.318	0.0035^a^		0.0052^b^
I	33	28	5	0.0028^d^	0.782 ± 0.721	0.0090^e^
II	36	30	6		0.918 ± 0.541	
III	17	7	10		1.312 ± 0.708	
IV	2	1	1		1.075 ± 0.813	
Tumor Grade		= 2.063	= 2.455	0.0678^a^		0.0061^b^
1	23	21	2	0.0882^d^	0.598 ± 0.429	0.0099^f^
2	25	17	8		1.009 ± 0.698	
3	37	25	12		1.112 ± 0.726	
Unknown	3	3	0			
Lymph Node Metastasis				0.1004^c^		0.0303^a^
Absent	38	32	6		0.833 ± 0.775	
Present	34	22	12		1.083 ± 0.612	
Unknown^†^	16	12	4			
Tumor Size (cm)		= 2.175	= 2.955	0.0283^a^	ρ = 0.303^b^	0.0060^b^
Median (Range)	2.2 (0.1 - 9.0)	2.0 (0.1 - 7.25)	2.5 (0.5 - 9.0)			
25th to 75th Quartile	1.18 - 3.00	1.0 - 2.5	1.7 - 4.0			
Lymphovascular Invasion				0.0720^c^		0.0201^a^
Absent	55	45	10		0.812 ± 0.599	
Present	32	20	12		1.177 ± 0.734	
Unknown	1	1	0			
ER Status				0.2514^c^		0.0489^a^
Positive	62	48	14		0.866 ± 0.594	
Negative	21	13	8		1.271 ± 0.822	
Unknown	5	5	0			
PR Status				0.0640^c^		0.1828^a^
Positive	58	47	11		0.864 ± 0.562	
Negative	28	17	11		1.142 ± 0.838	
Unknown	2	2	0			
HER-2/neu Status				0.0335^c^		0.1022^a^
Positive	19	10	9		1.181 ± 0.644	
Negative	61	49	12		0.923 ± 0.661	
Unknown	8	7	1			

^(a) ^Mann-Whitney, ^(b) ^Spearman Correlation, ^(c) ^Fisher's Exact Test, ^(d) ^Chi-Square Test, ^(e) ^Mann-Whitney with clinical stage dichotomized as stage I&II vs. III&IV, and ^(f) ^Kruskal-Wallis Test. Cases that were uninformative for a given variable were removed from statistical analysis. ^†^No lymphadenectomies were performed for these patients.

## Results

### MED28 levels are elevated in breast cancer

**Figure 2:**
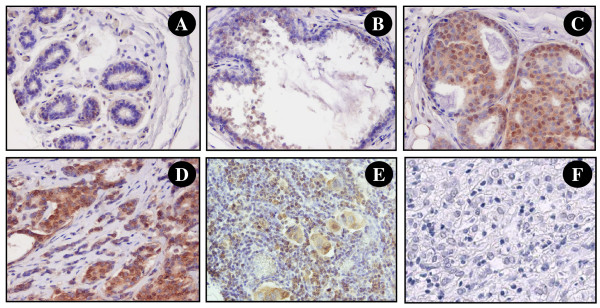
**MED28 expression in breast tissue samples**. By immunohistochemical staining, MED28 is observed in the cytoplasm and nucleus of epithelial cells. Shown is representative staining of **A) **morphologically normal tissue; **B) **ductal hyperplasia (DH); **C) **ductal carcinoma *in situ *(DCIS), **D) **invasive ductal carcinoma (IDC), **E) **lymph node metastases; and **F) **IDC negative control with non-immune primary antibody. In all experiments, substitution of primary antibody with a species matched non-immune reagent showed no staining.

Previously, expression of MED28 was found to promote proliferation in breast cancer cells [7]. Based on this, we predicted that MED28 expression levels could yield clinically relevant information about breast cancer development and/or progression. To start testing this, we examined the expression of MED28 in a population of women with breast cancer using high-throughput TMA technology. The TMA consisted of 242 surgical cases from patients seen at the UCLA Medical Center between 1995 and 2000. Table 1 shows the clinical and demographic characteristics of the patient population utilized for outcomes analyses. Consistent with previous results, MED28 expression could be observed in both the cytoplasm and nucleus of cells (Figure 2A, B, C, D, E, and 2F). The expression pattern in the nuclear and cytoplasmic components was highly correlated, therefore analyses of cytoplasmic expression are shown here, and analogous results examining nuclear staining can be found in the Additional Files (Additional File 1 and Additional File 2).

**Figure 3:**
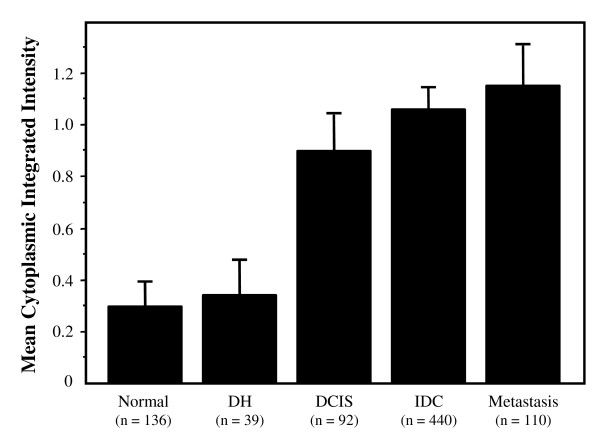
**MED28 expression by histology**. MED28 expression as classified by different TMA spot-level histologies. The columns show the mean integrated cytoplasmic expression of MED28; bars, SE. MED28 expression in DCIS, IDC, and metastatic lesions were significantly elevated compared to either normal or DH tissues (P < 0.0001). MED28 expression in DCIS lesions was also slightly different from expression in metastatic lesions (P = 0.040). n is the number of spots in each category.

We first examined the expression level of cytoplasmic MED28 for each histology or histopathology. We observed low expression of MED28 in morphologically normal and hyperplastic (DH) breast epithelium (Figure 3). In contrast, MED28 expression in DCIS and invasive ductal carcinoma lesions was approximately three-fold higher than either normal or DH levels (P < 0.0001; Figure 3). This confirms and validates previous findings showing enhanced expression of MED28 in breast cancer [6]. Similarly, malignant cells which had metastasized to the lymph nodes expressed >3 fold higher levels of MED28 than normal epithelium (P < 0.0001; Figure 3).

### Relatively high levels of MED28 predict a greater likelihood of tumor recurrence

**Figure 4:**
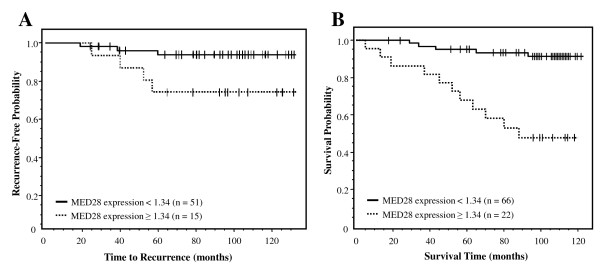
**MED28 expression levels predict tumor recurrence and survival in women with breast cancer**. Solid line is lower MED28 expression (cut-off < 1.34, integrated cytoplasmic expression); dashed line is higher MED28 expression (cut-off ≥1.34, integrated cytoplasmic expression). n is the number of individuals in each category. **A) **Higher MED28 expression predicted a greater risk of breast cancer recurrence following surgery (P = 0.027). **B) **Individuals with higher MED28 expression had an increased probability of death due to disease compared to those individuals with lower MED28 (P < 0.0001). In this group of individuals, 5 patients had no MED28 expression in their tumor and 83 patients had some degree of positivity.

We next examined whether MED28 expression yielded relevant information regarding disease outcome. First, we considered tumor recurrence. In a univariate Cox model, MED28 as a continuous variable was not a significant predictor of tumor recurrence (P = 0.113); however, when we dichotomized expression levels, MED28 was a significant predictor, with relatively higher levels of MED28 expression predicting a significantly greater chance of breast cancer recurrence than those with lower MED28 expression (Figure 4A; P = 0.027).

### Relatively high levels of MED28 predict a poorer survival outcome

**Figure 5:**
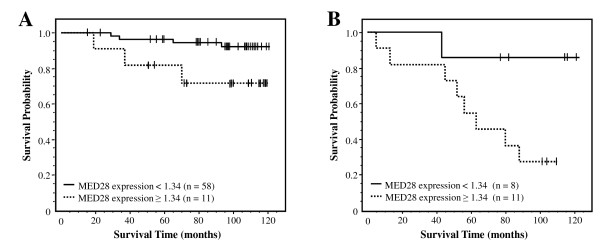
**MED28 levels predict survival likelihood in women with low stage or high stage breast cancer**. Solid line is lower MED28 expression (cut-off <1.34, integrated cytoplasmic expression); dashed line is higher MED28 expression (cut-off ≥1.34, integrated cytoplasmic expression). n is the number of individuals in each category. **A) **In women with low stage breast cancer (stage I/II), higher MED28 expression predicted a greater risk of death due to disease compared to those individuals with lower MED28 (P = 0.037). **B) **In women with high stage breast cancer (stage III/IV), higher MED28 expression predicted a greater risk of death due to disease compared to those individuals with lower MED28 (P = 0.034).

We next examined whether MED28 expression profile predicted survival. Importantly, MED28 expression both as a continuous variable (P = 0.011) and as a dichotomized variable (Table 2; P < 0.0001) was a significant predictor of disease-specific survival. As shown in Figure 4B, Kaplan-Meier analysis demonstrates that patients with relatively higher levels of MED28 had a dramatically lower probability of survival compared with individuals whose tumors expressed relatively lower levels of MED28 (P < 0.0001). This effect was observed both at early stage (Figure 5A; P = 0.037) and late stage (Figure 5B; P = 0.034) cancers.

**Table 2:** Univariate Cox Model Regression Analysis

Variable	Hazard Ratio	95% Confidence Interval	P-Value
Age at Diagnosis	0.976	0.937 - 1.02	0.250
Stage (I & II vs. III & IV)	5.81	2.16 - 15.7	<0.0001
Grade (I & II vs. III)	2.32	0.843 - 6.39	0.100
Lymph Node Metastasis	5.03	1.4 - 18.1	0.013
Tumor Size (cm)	1.19	0.926 - 1.52	0.180
Lymphovascular Invasion	3.04	0.951 - 9.7	0.061
ER Status	0.541	0.196 - 1.49	0.230
PR Status	0.562	0.209 - 1.51	0.250
HER-2/neu Status	3.71	1.25 - 11.1	0.018
MED28 expression	2.09	1.18 - 3.7	0.011

We further assessed any correlative association between MED28 expression and other clinico-pathological variables (Table 1). As a continuous variable, elevated MED28 was significantly associated with higher tumor grade (P = 0.006), lymph node metastases (P = 0.030), lymphovascular invasion (P = 0.024), and estrogen receptor (ER) negativity (P = 0.049). As a dichotomized variable, elevated MED28 was significantly associated with higher stage (P = 0.003), greater tumor size (P = 0.028) and HER2/neu negativity (P = 0.033). Because of these associations, we assessed whether MED28 was an independent predictor of disease survival. To do this we used a multivariate Cox model which included MED28 as a dichotomized variable, stage, tumor grade, age, ER expression status, and HER2/neu expression. MED28 emerged as an independent predictor of disease-specific survival (HR = 5.662, 95% CI = 1.178 - 27.21, P = 0.030; Table 3); MED28 was even a stronger predictor than stage (HR = 3.169, 95% CI = 0.821 - 12.23, P = 0.094).

**Table 3:** Multivariate Cox proportional hazards analysis

Variable	Hazard Ratio	95% Confidence Interval	P-Value
MED28 Dichotomized	5.662	1.178 - 27.21	0.030
Stage (I & II vs. III & IV)	3.169	0.821 - 12.23	0.094
Grade (I & II vs. III)	1.808	0.698 - 4.68	0.220
Age at Diagnosis	0.956	0.895 - 1.02	0.180
ER Status	0.832	0.241 - 2.87	0.770
HER-2/neu Status	1.346	0.399 - 4.54	0.630

## Discussion

In this study, we examined the expression of MED28 on a population basis using TMA technology. MED28 levels were increased in DCIS lesions as well as invasive breast cancer compared to morphologically normal breast epithelium. In addition, MED28 was up-regulated in metastatic cells in lymph nodes. These results are in agreement with previous results in which a smaller number of patient samples were examined [6]. Significantly, we further observed that MED28 was a strong predictor of disease outcome with higher levels of MED28 indicating an increased probability of death due to breast cancer in the 88 individuals examined with survival information. These results are consistent with data from *in vitro *and mouse model systems in which up-regulation of MED28 enhanced cell proliferation and tumor growth [7]. Inhibition of MED28 by antibody or siRNA blocked these effects [9].

The cellular function of MED28 is currently being elucidated. Although MED28 was initially discovered as a differentially expressed gene in human endothelial cells treated with conditioned media from human cancers [5], it was also characterized as a binding partner for the actin-associated neurofibromatosis 2 (NF2) tumor suppressor merlin as well as the adaptor protein Grb2 [12]. These and other results are consistent with MED28 functionally linking membrane receptor signaling to cytoskeletal changes. MED28 has further been shown to bind the SH3 domain of src-family members suggesting that one mode of operation is through interaction with kinase signaling molecules Src, Lck, and/or Fyn [11]. Interestingly, over-expression of MED28 *in vitro *has been shown to activate c-Src and stimulate the c-Src signaling pathway [8]. Src activation can contribute to the malignant phenotype through enhancing processes such as proliferation, invasion, migration, and metastasis (reviewed in [21-24]).

Interestingly, MED28 has been observed both in the cytoplasm and the nucleus as described by us and others [6,10,13]. Consistent with this, MED28 is one of the subunits of the highly conserved mammalian mediator complex. This complex functions as a co-activator required for the induction of transcription by RNA polymerase II [25-30]. It has been suggested that MED28 translocates between the nucleus and cytoplasm and therefore may potentially function in transducing membrane-derived signals into gene expression events.

## Conclusions

The present study shows that the expression of MED28 is relatively higher in both early and advanced breast cancer lesions. Such elevated levels predict a poorer survival. That MED28 expression was elevated in DCIS lesions compared to normal and was predictive in early as well as late stage patients suggests that alterations in the MED28 signaling axis may be an early indicator of disease progression and a potential therapeutic target. In addition to its potential usefulness as a prognostic factor, MED28 may eventually prove useful for targeted therapy: a recent study showed that inhibition of MED28 resulted in smaller breast tumor xenografts in mice [9].

## Abbreviations

TMA: Tissue Microarray; DH: ductal hyperplasia, DCIS: ductal carcinoma *in situ*, ER: estrogen receptor; PR: progesterone receptor, EG-1: endothelial-derived gene-1; HR: hazard ratio; CI: confidence interval; NF2: neurofibromatosis 2.

## Competing interests

The authors declare that they have no competing interests.

## Authors' contributions

NKY: performed experimentation, statistical analyses and data interpretation. ELM: aided in statistical analyses and data interpretation and drafted the manuscript. YE: performed scoring of the tissue array. AL and SH: provided expertise in statistical analysis. DBS and DC: aided in project strategy and study planning. LG: aided in data interpretation and project strategy, planning, and design and critically revised the manuscript. All authors have read and approved the final manuscript.

## Pre-publication history

The pre-publication history for this paper can be accessed here:

http://www.biomedcentral.com/1471-2407/10/335/prepub

## Supplementary Material

Additional file 1 **Correlation of Nuclear and Cytoplasmic Expression of MED28**. MED28 expression was observed in both the cytoplasm and the nucleus in most breast tissues examined. To assess correlation, integrated intensity measures for each spot were compared for nuclear versus cytoplasmic expression. As shown in File 1S, there is a high degree of correlation between the expression pattern in the nuclear and cytoplasmic components. Click here for file

Additional file 2 **Nuclear Expression of MED28 is Predictive of Survival in Breast Cancer Patients**. Similar to our analyses using cytoplasmic MED28 expression levels, we also examined whether the level of nuclear MED28 expression predicted survival outcome in women with breast cancer. Case expression levels were pooled as previously described [16-19], and patient integrated MED28 expression levels were dichotomized into high versus low MED28 levels using an optimized cut-point. Survival was visualized via Kaplan-Meier curves, and survival differences were tested using the log-rank test as described in this manuscript and as previously outlined [16-20]. Similar to results based on cytoplasmic expression, higher levels of nuclear MED28 predicted a much poorer survival (File 2S; P = 0.0047) Click here for file
