# Supplementary figures and images for: Elevated MED28 expression predicts poor outcome in women with breast cancer
Source: BMC Cancer. 2010 Jun 28;10:335. doi: 10.1186/1471-2407-10-335 (PMC2907343; doi:10.1186/1471-2407-10-335)

## Slide 1
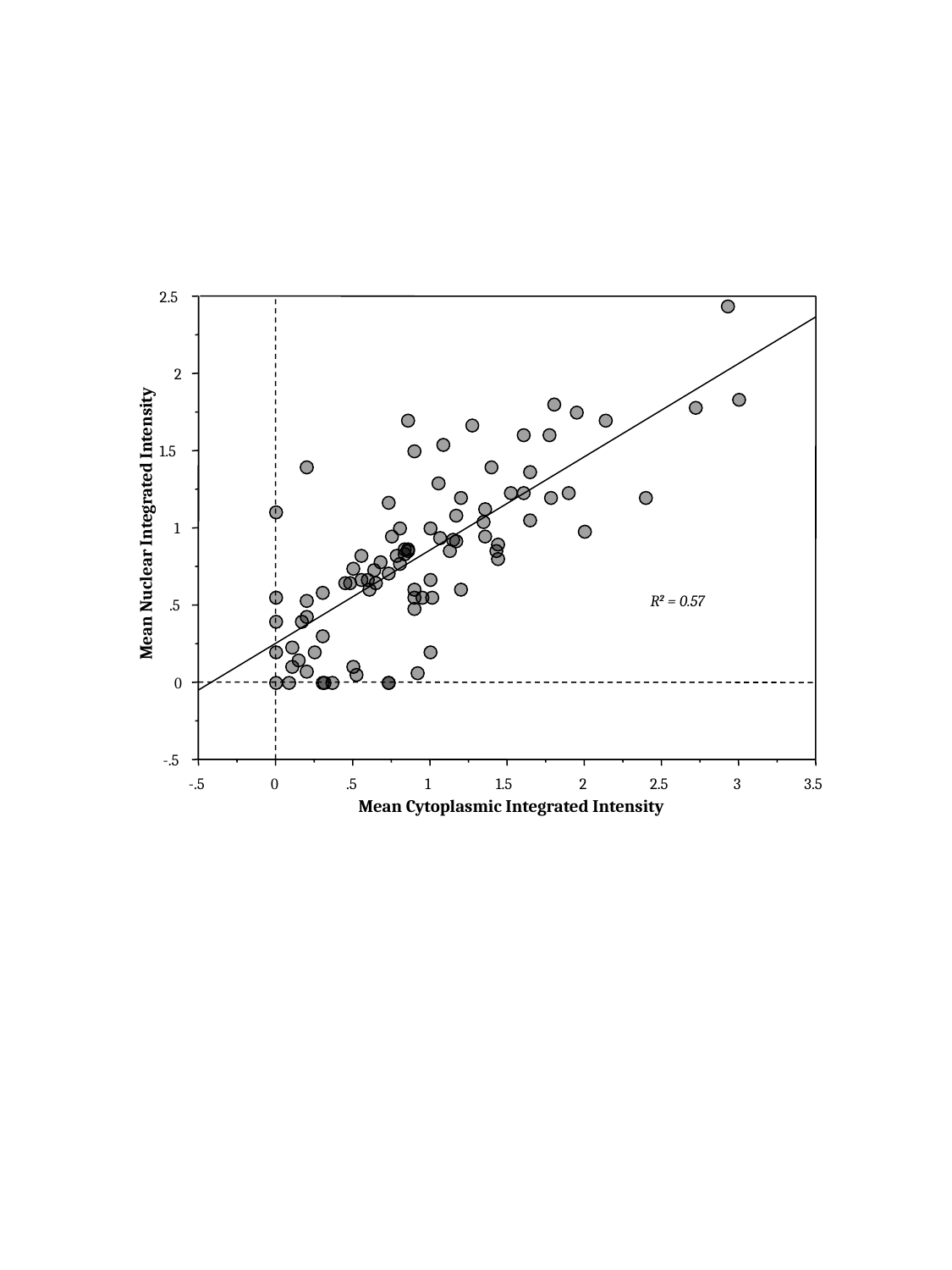

2.5
2
1.5
Mean Nuclear Integrated Intensity
1
R2 = 0.57
.5
0
-.5
-.5
0
.5
1
1.5
2
2.5
3
3.5
Mean Cytoplasmic Integrated Intensity

Supplement: Additional file 1 — Correlation of Nuclear and Cytoplasmic Expression of MED28. MED28 expression was observed in both the cytoplasm and the nucleus in most breast tissues examined. To assess correlation, integrated intensity measures for each spot were compared for nuclear versus cytoplasmic expression. As shown in File 1S, there is a high degree of correlation between the expression pattern in the nuclear and cytoplasmic components. [file 1471-2407-10-335-S1.PPT]

## Slide 1
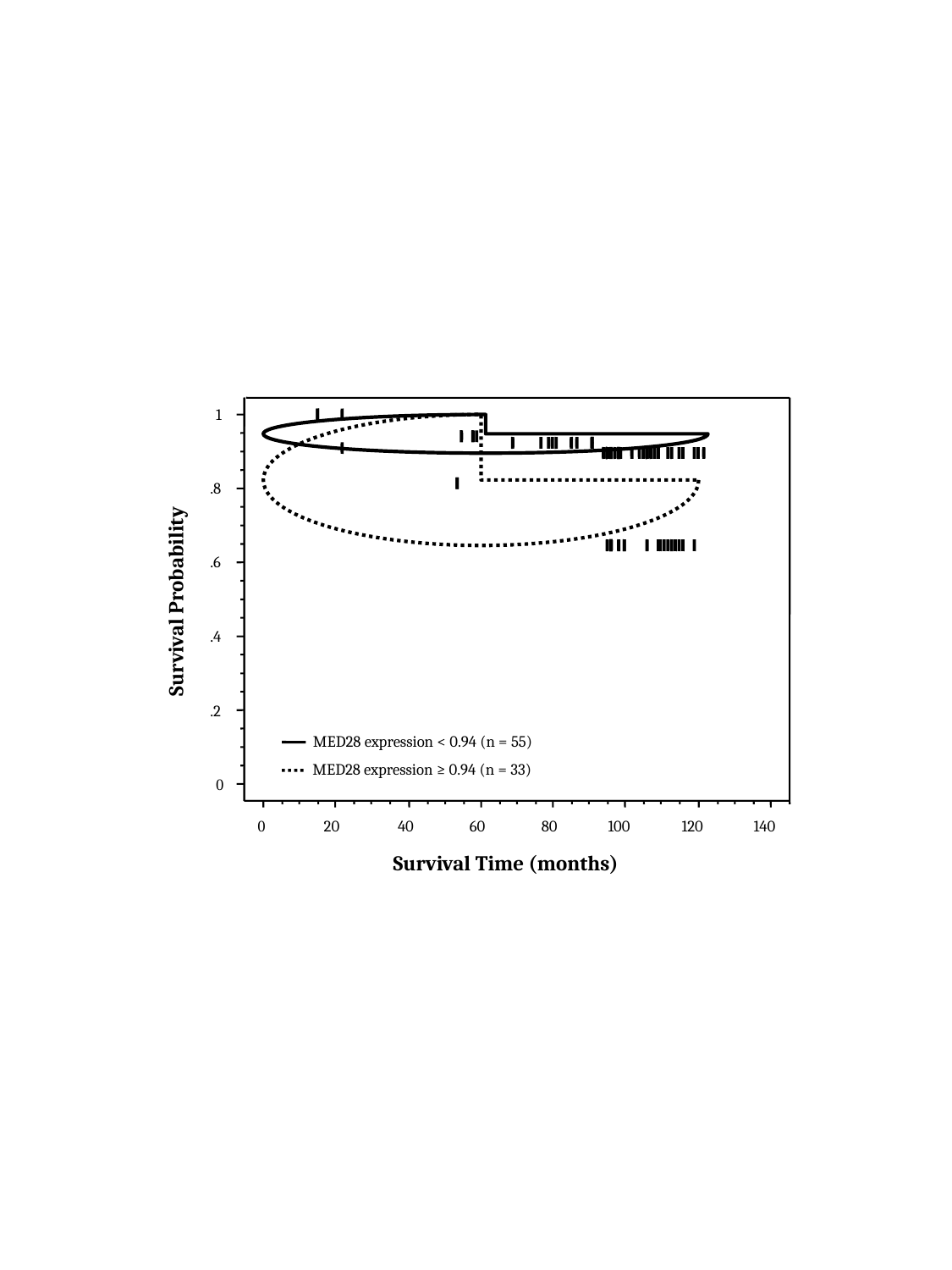

1
.8
.6
.4
.2
0
0
20
40
60
80
100
140
120
Survival Probability
Survival Time (months)
MED28 expression < 0.94 (n = 55)
MED28 expression ≥ 0.94 (n = 33)

Supplement: Additional file 2 — Nuclear Expression of MED28 is Predictive of Survival in Breast Cancer Patients. Similar to our analyses using cytoplasmic MED28 expression levels, we also examined whether the level of nuclear MED28 expression predicted survival outcome in women with breast cancer. Case expression levels were pooled as previously described [16-19], and patient integrated MED28 expression levels were dichotomized into high versus low MED28 levels using an optimized cut-point. Survival was visualized via Kaplan-Meier curves, and survival differences were tested using the log-rank test as described in this manuscript and as previously outlined [16-20]. Similar to results based on cytoplasmic expression, higher levels of nuclear MED28 predicted a much poorer survival (File 2S; P = 0.0047) [file 1471-2407-10-335-S2.PPT]
